# Characteristic of intoxicated cyclists compared to sober cyclists admitted to a London Major Trauma Centre

**DOI:** 10.1186/1757-7241-23-S2-O4

**Published:** 2015-09-11

**Authors:** Anna E Forbes, John M Schutzer-Weissmann, Alex Wang, Matthew Wordsworth, Mark H Wilson

**Affiliations:** 1 Intensive Care Unit and Major Trauma Ward, St Mary's Hospital, Imperial College Healthcare NHS Trust, London, UK

## Background

Studies have suggested that alcohol is an independent risk factor for cycling accidents [1].

This study aimed to compare mechanisms of injury, injury patterns and helmet use in intoxicated and sober cyclists.

## Methods

Cyclists admitted to a London Major Trauma Centre were identified from Emergency Department Records, the Trauma audit and research network, Major Trauma Ward Lists and intensive care admission documents.

Admission toxicology and prehospital and emergency department documentation were analysed to identify patients who had negative alcohol tests and those who had elevated blood alcohol levels or a history of recent alcohol ingestion. Lack of a history of alcohol use was not used to identify sober cyclists. These patient groups were then analysed to assess helmet use and injury pattern.

## Results

Initially 186 cyclists were identified (152 males and 28 females) and of these 80 had alcohol levels or positive history recorded. Of the 186 cyclists 10% of female cyclists were intoxicated compared to 20% of males. In the intoxicated group (n=32) fall was the most common mechanism of injury (53%) whereas in the sober group (n=46) collision with a car was the most common (52%). In those for whom helmet wearing data was available (17 intoxicated, 36 sober) 82% of drunk cyclists weren't wearing a helmet compared to 55% of sober cyclists. Injury patterns were similar in both drunk and sober groups.

## Discussion

This study was limited by the lack of toxicology data available. However it showed that intoxication was present in at least 18% of those admitted to a major trauma centre following cycling accidents. It was also identified that intoxicated cyclists were less likely to protect themselves with a helmet and in contrast to sober cyclists were less likely to have accidents involving other road users.
